# Magnetic Seizure Therapy for Unipolar and Bipolar Depression: A Systematic Review

**DOI:** 10.1155/2015/521398

**Published:** 2015-05-13

**Authors:** Eric Cretaz, André R. Brunoni, Beny Lafer

**Affiliations:** ^1^Service of Electroconvulsive Therapy, Department and Institute of Psychiatry, University of São Paulo, Dr. Ovídio Pires de Campos Street 785, 05403-903 São Paulo, SP, Brazil; ^2^Bipolar Disorder Research Program, Department and Institute of Psychiatry, University of São Paulo, Dr. Ovídio Pires de Campos Street 785, 05403-903 São Paulo, SP, Brazil; ^3^Service of Interdisciplinary Neuromodulation (SIN), Laboratory of Neurosciences (LIM-27), Department and Institute of Psychiatry, University of São Paulo, Dr. Ovídio Pires de Campos Street 785, 05403-903 São Paulo, SP, Brazil

## Abstract

*Objective*. Magnetic seizure therapy (MST) is a novel, experimental therapeutic intervention, which combines therapeutic aspects of electroconvulsive therapy (ECT) and transcranial magnetic stimulation, in order to achieve the efficacy of the former with the safety of the latter. MST might prove to be a valuable tool in the treatment of mood disorders, such as major depressive disorder (MDD) and bipolar disorder. Our aim is to review current literature on MST. *Methods*. OVID and MEDLINE databases were used to systematically search for clinical studies on MST. The terms “magnetic seizure therapy,” “depression,” and “bipolar” were employed. *Results*. Out of 74 studies, 8 met eligibility criteria. There was considerable variability in the methods employed and samples sizes were small, limiting the generalization of the results. All studies focused on depressive episodes, but few included patients with bipolar disorder. The studies found reported significant antidepressant effects, with remission rates ranging from 30% to 40%. No significant cognitive side effects related to MST were found, with a better cognitive profile when compared to ECT. Conclusion. MST was effective in reducing depressive symptoms in mood disorders, with generally less side effects than ECT. No study focused on comparing MST to ECT on bipolar depression specifically.

## 1. Introduction

### 1.1. Mood Disorders and ECT

Mood disorders, such as major depressive disorder (MDD) and bipolar disorder (BD), are highly prevalent and debilitating conditions, associated with high suicidality rates, elevated treatment costs, and heavy social and economic burden [1]. The treatment of depressive episodes is associated with a 30–40% rate of nonresponse [2]. Few effective treatments are currently used and approved for resistant unipolar and bipolar depression.

Electroconvulsive therapy (ECT) is regarded as the most effective treatment for depression [3], in both BD and MDD, with remission rates ranging from 50% to 75% [4–6]. ECT is also effective for manic episodes, with reported remission rates of up to 85% [7–11].

Despite its high effectiveness, the use of ECT is limited by its side effects, especially the development of cognitive deficits and, in particular, memory impairment [12–16]. Such side effects can range from mild or nonexistent in some patients up to severe and distressing in others [14, 17–19]. Of those, memory loss is the single most often cognitive complaint reported by patients undergoing ECT [12]. The cognitive effects of ECT have been shown to be dependent upon numerous ECT parameters, namely, electrode placement, frequency of sessions, electric dose, and pulse width [14, 17, 20].

### 1.2. Magnetic Seizure Therapy

The search for improved ECT techniques, which could be able to reduce or minimize memory-related side effects whilst maintaining efficacy, has been the subject of a considerable amount of investigation [21]. However, the use of an electrical stimulus to induce seizures is a fundamental limitation in refining convulsive therapy and limiting its side effects profile [22]. Control over the spatial distribution and magnitude of intracerebral current density is limited by high skull impedance, which shunts most of the electrical stimulus through the scalp and cerebrospinal fluid (CSF) and away from the brain. The substantial impedance of the scalp and skull means that the bulk of the electrical stimulus is shunted away from the brain, resulting in widespread stimulation of cortical and subcortical regions. There are also individual differences in skull anatomy that result in uncontrolled variation in intracerebral current density [23]. Measurements in humans of shunting across the scalp and skull range from 80% up to 97%. Recent studies with computer models further support the notion that current dissipation in ECT is considerable and that the electrode placement associated with more severe cognitive side effects, bitemporal (BL-ECT), is also the placement with higher shunting and with deeper brain stimulation [24].

The possibility of noninvasive stimulation of specific areas in the cerebral cortex through magnetic stimulation was first demonstrated in 1985 [25]. The use of transcranial magnetic stimulation (TMS) has since been studied as a treatment for depression, and a number of meta-analyses and Sham-controlled trials have confirmed that it is associated with statistically significant antidepressant effect [26]. TMS employs a rapidly changing magnetic field to induce an electrical current in a targeted brain region, most often the left dorsolateral prefrontal cortex (DLPFC) [27]. Magnetic stimulation is more focal than electrical stimulation because it avoids the impedance of the scalp and skull and results in an induced electric field confined to superficial cortex. Thus, magnetic stimulation has allowed more control over current paths and current density within cortical tissue [22, 28] and grants TMS a safer, well-tolerated side effects profile [27]. However, TMS efficacy in treatment-resistant patients is limited. A recent meta-analysis [26] has shown that repetitive transcranial magnetic stimulation (rTMS), despite significantly reducing depressive symptoms, is not as effective as ECT for the treatment of refractory depression, leading to a mean reduction of 9.3 points on the Hamilton Depression Rating Scale (HDRS), while ECT leads to a reduction of 15.42 points. Also, ECT is reported to be more effective in reducing suicidality than rTMS [26].

It has long been known that TMS could induce seizures. Such phenomenon was initially considered to be accidental and regarded as a complication of method [29–31]. However, the possibility of intentionally inducing seizures by magnetic pulses has been formally proposed as early as 1994 by Sackeim [32]. Once rTMS had been found to have significant antidepressant effects at subconvulsive levels, and considering the superior antidepressant potency of ECT, it has been hypothesized that, under controlled conditions in a patient under anesthesia, increasing the magnetic stimulus into the convulsive range the resultant seizure could confer robust antidepressant properties as seen with ECT [33]. It was also hypothesized that the more accurate and focal seizures triggered by magnetic stimulation could lead to less adverse effects than seizures triggered by ECT, while retaining its therapeutic capacity [22].

The first published report of seizures successfully and deliberately induced by rTMS dates back to 2001, described by Lisanby et al. [23]. Two exemplars of* Macaca mulatta* were chosen for the experiments because of brain-to-coil size ratio that was closer to humans when pediatric sized coils were used and for their ability to perform cognitive tasks with which to access the potential cognitive side effects of the method [34]. The animals were subjected to rTMS sessions under general anesthesia, using both a commercially available TMS device (MAGSTIM Super Rapid, Magstim Company Ltd., Whitland, Wales) and a customized MAGSTIM device, with eight booster modules instead of the usual four modules, capable of broader pulse width and yielding 40% more power per pulse. The two trials conducted with the commercial MAGSTIM device failed to trigger seizures in the primates. The custom device, however, was capable of inducing seizures in both subjects in all trials, therefore illustrating the need for more powerful stimulation than a standard TMS device could deliver in order to elicit seizures.

Further studies comparing electroconvulsive shock (ECS), the animal equivalent of ECT, with MST and Sham on non-human primates were conducted in order to assess the safety of the procedure. Results pointed that MST has significantly less impact than ECS on functions such as spatial memory, time to task completion, and anterograde memory. In fact, there was no difference between MST and Sham, even when with the use of higher-intensity stimulus, with pulse frequency of up to 100 Hz [35–37]. Postmortem studies conducted on the experimentation animals did not reveal morphologic changes or histological lesions [38, 39].

Soon after the first description of MST in animals, initial human reports were published. In 2001, Lisanby et al. [40] described the first use of MST in a 20-year-old patient with treatment-resistant episode of MDD, who had undergone for the previous three years several pharmacological trials, with different classes of antidepressant drugs, without success, being referred to ECT. The patient received four sessions of MST before following up with conventional ECT. Generalized tonic-clonic seizures were successfully elicited in the four MST treatments, with Hamilton Depression Rating Scale scores decreasing from 20 at baseline to 13 after the fourth session. No severe adverse effects were reported and Mini Exam of Mental State scores remained unaltered throughout the trial.

In 2003, Kosel et al. [41] reported a second patient treated with MST for treatment-resistant MDD. This time, a 66-year-old patient was submitted to a full trial of MST, with 12 sessions, until remission was achieved. Hamilton Depression Rating Scale (HDRS) scores dropped from 33 to 6 and Beck Depression Inventory (BDI) dropped from 40 to 11. The sessions were well-tolerated, with no somatic or cognitive complaints. A more comprehensive cognitive assessment battery was employed, and the patient did not show any significant cognitive deficits following MST. Actually, there was an improvement on some cognitive tests following treatment.

Since the mechanism of action of MST involves the induction of seizures, the use of general anesthesia with muscle relaxation and clinical support is necessary, much like in ECT. The use of a bite block, which is mandatory for patients receiving ECT, on the other hand, is not necessary since there is no direct stimulation of the masseter muscle by shunted electric current. However, the loud “clicking” noise causing the coil might require the use of earplugs by both patients and staff. Different coils have been tested, with the nonfocal round coil and the double cone coil reported to be reliable in seizure induction, while the more focal “figure of 8” coil was considered inefficient in inducing seizure. Another concern related to the coils is the heating of the equipment, which is more pronounced than in TMS coils and required previous cooling and the use of heat-resistant materials [34].

The seizure induced by MST is quite different from that produced by ECT. Magnetic pulses, such as those employed by rTMS and MST, are capable of focusing the stimulation to a specific area, since they pass unhindered into the brain without resistance and are not shunted through scalp, skull, and CRL like ECT's electric stimulus. On the other hand, magnetic pulses generated by most commercially available coils only penetrate a few centimeters deep, while electric current can reach deeper structures more easily. Therefore, a major difference between MST and ECT is the former's capacity to focus stimulation, with MST-induced seizures originating on superficial regions of the cortex, unlike ECT, where electrical current passes deep through the brain [22]. Consequently, it is possible that MST may produce similar therapeutic benefits to ECT without inducing memory-related side effects, as there is no direct electrical stimulation of medial temporal lobe structures, such as the hippocampus, which are implicated in ECT-related memory impairment [42].

## 2. Aims

Our objective was to review current clinical evidence on magnetic seizure therapy, its effectiveness on unipolar and bipolar depression, and its side effects profile, with special emphasis on the cognitive aspects. Whenever possible, comparisons with ECT were drawn in order to establish similarities and differences between the methods.

## 3. Methods

### 3.1. Literature Review

In order to systematically review the current literature on the use of magnetic seizure therapy in the treatment of mood disorders in accordance with the PRISMA guidelines, the authors performed searches in the Ovid and MedLine databases using the following terms: (1) “Magnetic Seizure Therapy” OR (2) “Magnetic Seizure Therapy” AND Depression OR (3) “Magnetic Seizure Therapy” and Bipolar. Articles dating from 1985, the year the first papers on Transcranial Magnetic Stimulation were published, to August 2014 were selected. The results were managed with Mendeley Desktop (version 1.10.1.0, © Mendeley, Ltd.) software.

### 3.2. Eligibility Criteria

The inclusion and exclusion criteria for studies were as follows: inclusion: (a) manuscript in English; (b) clinical trials, either open-labeled or blinded studies; (c) studies on human subjects and exclusion: (a) virtual models; (b) animal studies; (c) case reports.

### 3.3. Studies Overview

Our initial search yielded 75 references, 64 of which were initially excluded, based on eligibility criteria, leaving 11 articles. In a subsequent analysis, 3 references were excluded after the abstracts were reviewed and did not meet eligibility criteria. Ultimately, 8 studies were included.

### 3.4. Data Extraction

The following variables were extracted per a structured checklist that we developed: (a) overview: study design, authors, year of publication, technique, summary, and other relevant data; (b) demographics: total sample (number) and intervention groups; (c) assessment of mood disorder: method of diagnosis (clinical or structured interviews); and (d) outcomes: description of each study's results.

### 3.5. Quality Assessment

To assess the methodological heterogeneity between studies, each report was evaluated with regard to quality, focusing on 2 critical methodological issues: (a) internal validity: for clinical studies, whenever possible the authors followed the Cochrane guidelines to determine the risk of bias in randomization (selection bias), blinding and control comparison (performance bias), and outcome assessment and reporting (attrition, measurement, and reporting biases); however, since a small number of double-blind randomized clinical trials were found, open-label studies were included as well; (b) construct validity: we determined whether the operational criteria for mood disorders were appropriate, that is, whether each study fulfilled the following criteria: (i) clinical studies that focused on MST and (ii) articles on mood disorders.

## 4. Results

A total of eight studies were identified by our search. A summary of results is available on Table 1. Outcomes on clinical improvement and cognitive side effects are described separately below.

### 4.1. Clinical Improvement

The first clinical trial assessing MST's effectiveness compared to ECT in reducing depressive symptoms was reported by White et al. [43]. Depressive symptoms were evaluated with the HDRS before and after the series of 10 to 12 treatments. Researchers reported that both methods resulted in a decrease in depressive scores, and ECT reduced mean HDRS from 30 at baseline to 6 after treatment, while on the MST group mean HDRS at baseline was 32, dropping to 14 at the end of the trial. Results suggested that while MST demonstrated a safer cognitive profile, its effectiveness was still below ECT. Only 53% of the patients treated with MST achieved a 50% or greater improvement on the depression rating scores. Considering that ECT responses have been previously associated with electric dose in relation to the seizure threshold, a possible explanation for MST's lower efficacy could be the fact that treatments were delivered only on average 1.3 times above magnetic seizure threshold. The authors suggested that better response rates could have been achieved with higher stimulation intensity; however, MST devices available at the time could only produce short trains of 8 seconds and 50 Hz at maximum output.

In an attempt to improve effectiveness of MST, it has been proposed that more intense stimuli could be necessary, which could be achieved by employing higher frequency, up to 100 Hz, and longer trains. Such techniques became known as high-dose MST (HD-MST). The first comparison between effectiveness of HD-MST and ECT was published in 2011 by Kayser et al. [44] in patients on a treatment-resistant depressive episode of BD and MDD. Primary outcome measure was response, defined by 50% reduction of Montgomery and Asberg Depression Rating Scale (MADRS) or remission, defined by MADRS score of less than 10. Other clinical measures included HDRS scores, Hamilton Anxiety Scale (HAMA), BDI, and the 90-Item Symptom Checklist (SCL-90). Out of ten patients on the MST group, six were responders, three of which achieved remission. In the ECT group, four patients were responders, all of which achieved remission. No significant difference in effectiveness of MST and ECT was found. It is noteworthy that ECT procedures employed by the researchers differed from the usual practice inasmuch as low down RUL-ECT was used, which reportedly could be less effective than high-dose RUL-ECT.

Further investigation of HD-MST and its impact on depressive symptoms was reported by Fitzgerald et al. in 2013 [45]. Clinical assessment was done with the MADRS, the primary outcome variable, as well as the 17-item HAM-D, BDI, BPRS, and the CORE rating of melancholia. Response was defined as a reduction of 50% in the MADRS. Scores were rated at baseline, after six sessions of MST and after the end of treatment. Out of 13 patients, five patients met response criteria, of which two achieved remission. One patient dropped out of the study due to a desire to receive ECT. Average MADRS scores dropped from 39.0 to 26.5, HAMD decreased from 26.7 to 19.2, BDI went from 35.8 to 26.7, and BPRS dropped from 20.7 to 13.6. No differences were detected between responders and nonresponders. The authors note that the response to MST in this sample was significantly inferior to the reported effectiveness of ECT, which generally yields remission rates of up to 80%.

The first investigation of regional glucose brain metabolism in MST-treated patients was reported by Hoy et al. in 2013 [46]. Ten patients suffering from MDD were selected for an open-label study. Patients received high-dose MST three times a week, and clinical measures were performed at baseline, after every 6 treatments and at the end of the MST treatment. The primary clinical outcome variable for response was MADRS score, and response was defined as 50% reduction on the score, while reduction of 20–50% was considered partial response and less 20% nonresponse. Cognitive functions were assessed. Fluorine-18-labeled deoxyglucose (FDG) PET/CT was performed in the week preceding the first MST treatment and a few days (average 3.8) after completion of the MST treatment course. Out of ten patients, four were considered responders. Average MADRS scores dropped from 40 at the baseline to 27.2 at endpoint. Neuropsychological assessment showed no cognitive adverse effects to the treatment. Significant relative glucose metabolism increases were seen in the globus pallidus, substantia nigra, putamen, and the orbital frontal cortex. Other findings, though not at the same level of significance, include increases in the glucose metabolism of the medial frontal cortex and the dorsolateral prefrontal cortex. There were no significant overall decreases in activity after MST. Differences between responders and nonresponders were only significant at a trend level and no relation was found between changes in relative glucose metabolism and changes in depression scores. Authors ponder that such findings could be due to the small sample.

### 4.2. Cognitive Side Effects

In 2003, Lisanby et al. reported the first series of patients submitted to MST [22]. A total of 10 patients suffering from MDD and referred to an ECT course were selected for a randomized, within-subject trial. Patients were blind to which treatment, MST or ECT, they would receive. The first two sessions of each patient were used to determine seizure threshold for both MST and ECT, followed by two sessions of suprathreshold stimulation with MST and ECT, and finally ECT sessions for the remaining treatments. Due to the high-frequency clicking noise intrinsic to magnetic stimulation, earplugs were worn by patients during both MST and ECT sessions. A bite-block was inserted immediately prior to seizure elicitation to protect the teeth, even if MST did not produce the marked jaw contraction as typically seen with ECT, since no current passes through the masseter. MST was administered with a modified Magstim stimulator, with a pulse width of 0.5 ms and 60 Hz at 100% output. All suprathreshold MST sessions were all given at maximal stimulator output (400 pulses). Tonic-clonic seizures similar to those seen with conventional ECT were elicited on all MST sessions, albeit seizure duration was shorter on average for MST (40.9 s on threshold and 49.5 s on suprathreshold stimulation) compared to ECT (101.0 s on threshold and 74.4 s on suprathreshold stimulation). A neuropsychological battery sampling multiple cognitive domain was administered by a blind rater at baseline and immediately before and after each of the four test sessions, evaluating cognitive functions such as memory, orientation, and attention, as well as the Squire Memory Test (Sentences), the Buschke Selective Reminding Test, and a Complex Figure Test. Patients had fewer subjective side effects and recovered orientation more quickly with MST than ECT. MST was also superior to ECT on measures of attention, retrograde amnesia, and category fluency.

To further investigate the cognitive side effects of MST compared to ECT, White et al. [43] conducted a study evaluating the amount of time patients took to recover orientation after each procedure, as well as reduction of depressive symptoms scores. Ten patients with MDD were submitted to MST and results compared to other 10 case-matched patients undergoing ECT treatment in an open-parallel study design. All patients underwent a series of 10–12 treatments of either MST or ECT. Time to recover orientation was assessed by a blinded observer in the postanesthesia recovery area who asked simple questions such as name, place, and day of the week after each session. Orientation recovery time was considerably shorter on the MST group, averaging 4 minutes, while patients submitted to ECT took an average of 18 minutes to correctly answer the questions. Such results suggested that MST could present a safer cognitive profile compared to ECT.

The first report of HD-MST on humans was published in 2008 by Kirov et al. [47]. Eleven patients suffering from TR MDD were enrolled for a within-subject open study comparing orientation recovery time of a single ECT and a single HD-MST session. Recovery of orientation after treatments was assessed by asking the patient for their name, date of birth, age, place, and day of the week. The point of orientation recovery was defined as the time when a patient was able to recall four of these five items. Recovery of orientation was much faster after MST than after ECT. The mean time to recovery after successful seizures elicited by MST was 7 min 12 s, against 26 min 35 s for ECT. The authors did not report effectiveness data on this study, since this was not the study focus and only one MST session was performed on each patient.

Another study, this time comparing the effectiveness, electrophysiological characteristics, and cognitive side effects of HD-MST and ECT, was published in 2011 by Kayser et al. [44]. Twenty patients in a treatment-resistant depressive episode were enrolled and blindly randomized in two groups of ten, treated with either ECT or MST. It is noteworthy that for the first time patients suffering from bipolar disorder were included in a MST trial, two patients with Type II BD receiving ECT and one Type I and one Type II patients on the MST group. A neuropsychological battery was employed before and after treatment to evaluate such cognitive domains as memory, learning, executive function, language, and processing speed. Also, after each session patients were assessed immediately in order to evaluate orientation. Recovery was defined as the time when patients opened their eyes and breathed independently, while reorientation time was assessed by asking the patient for her/his names, date of birth, age, place, and day of the week. Recovery and reorientation were faster in the MST group, at 1 min 42 s and 2 m 16 s respectively, compared to the ECT group, with recovery at 4 min 3 s and reorientation at 8 min 21 s. Except for that, no significant differences in cognitive side effects were found between groups. EEG activity was similar in both groups, consisting of high-amplitude synchronized theta activity and equal postictal suppression. However, on the MST group seizure duration was briefer and some patients showed delayed ictal EEG activity and duration of motor and ictal activity in MST-treated patients had about the same length. It is important to notice that the ECT procedures in this paper are quite different from other studies [5]. The authors used right unilateral electrodes (RUL-ECT) but chose to apply stimulus three times above seizure threshold, which is reported to be less effective than high-dose RUL-ECT. On the other hand, lower electrical doses are associated with less cognitive impairment than high-dose ECT, which could explain the absence of significant cognitive side effects on the ECT group.

Kayser et al. further explored seizure characteristics and cognitive aspects of HD-MST compared with ECT [48]. Seven patients in a treatment-resistant depressive episode were enrolled, six suffering from MDD and one from bipolar disorder Type II. Such patients had failed to respond to a 12-session MST course and were then referred to ECT 12 ECT sessions in an open-label, within-subject trial. None of the patients responded to the 12 sessions of ECT. Reorientation time was, once again, shorter, shorter after MST than after ECT. Except for the fact that seizures lasted longer on patients receiving ECT, the authors found no significant differences in visible motor seizures and EEG activities between the two procedures, including postictal suppression, a measure reported to predict response to ECT.

In 2013, Fitzgerald et al. [45] reported an open-label trial in which 13 patients with TR-MDD received MST, assessing its effectiveness and side effects. MST was applied three times per week, each patient receiving up to 18 treatments. A comprehensive neuropsychological battery was employed [47], and patients were evaluated at baseline, after six sessions and lastly after the end of treatments. Out of 13 patients, 12 completed the trial; the remaining subject chose to abandon the study due to poor response and desire to receive ECT. Average time to reorientation was 82.8 seconds. In fact, the fast recovery from the procedure might have created an unexpected side effect: four patients awoke from the procedure while still under effect of muscle relaxation. Neuropsychological tests showed no cognitive impairment after MST; in fact nonsignificant improvements were detected in several domains. One patient reported severe headaches. Unlike previous reports [44, 48], the authors reported EEG patterns unlike those expected on ECT patients. Not only were the motor seizures shorter, but also ictal activity was of less amplitude and there was typically much less postictal suppression. In some patients, ictal activity was not always apparent on the EEG despite a clear motor seizure. The authors also note that the response to MST in this sample was significantly inferior to the reported effectiveness of ECT, which generally yields remission rates of up to 80%.

The cognitive side effects of MST were recently explored by Polster et al. [49], who compared its impact on acute memory retrieval to ECT. Twenty patients suffering from treatment-resistant MDD were randomly assigned to two groups, one treated with MST and the other with ECT. A further ten healthy controls were included for comparison with the treatment groups. Memory assessment consisted of a customized test. On each of the 2 treatment control days and 2 treatment-free control days, the patients were given 3 consecutive learning trials in the morning to learn 40 words. Words were clustered into pairs and assigned to a hypernymic category for additional differentiation between storage or retrieval disruption of memory. This enabled the recording of cued recall providing information about the category. After treatment, patients were initially asked to remember all 40 of the words by themselves (delayed recall). Subsequently, they were provided with the name of each hypernymic category to enable them to recall all 40 words independently from delayed recall, again. The authors point that if patients extraordinarily benefit from these cues, this is indicative of a retrieval-based rather than a storage-focused memory disruption. By comparing memory performance on treatment days to control days, treatment-induced memory disruption was evaluated. To enable evaluation of treatment caused effects on a particular subject, the patients were treated with MST or ECT on 2 of the 4 testing days, whereas the other 2 days served as control. After ECT, delayed recall was disturbed, whereas after MST, it was not. However, this difference in performance was no longer apparent upon cue application.

## 5. Discussion

The results of this review show that MST is as effective as ECT in inducing generalized tonic-clonic seizures, both in animals and in humans. However, there are considerable differences in the mechanism of induction, since magnetic pulses are not shunted by the scalp and skull, unlike ECT's electric current, allowing for a more focused and superficial stimulation, which on its turn might have implications for its electrophysiological aspects, effectiveness, and side effects. MST-induced seizures are shorter lasting. In most animal studies and some clinical studies MST also led to less postictal suppression and less EEG amplitude compared to ECT-induced seizures, although two studies reported similar EEG characteristics for both groups [44, 48]. Such results could possibly be related to the use of RUL-ECT and low electric dose, which are associated with less cognitive loss but less effectiveness as well.

The clinical studies so far reported all support that MST is an effective treatment for depressive episodes, with response rates ranging from 40% to 60% and remission rates ranging from 15% to 30%. It is noteworthy that most trials were conducted on patients suffering from TRD, who had failed previous therapeutic strategies and therefore had a worse prognosis. On the other hand, such results are still far from those expected from ECT, which is reported to lead to remission rates of 50% to 70% on the same conditions. The results could be related to the parameters of MST, such as frequency of pulses and total pulse count. High-dose MST (HD-MST) is an attempt to bridge this gap, and further studies are being conducted in order to improve its effectiveness.

Both the neurocognitive effects and the effectiveness of ECT are related in part to the stimulus parameters with higher doses conferring increased adverse effects but perhaps better response rates on the other hand. For example, 6 × seizure thresholds RUL-ECT can achieve results similar to BL-ECT [50–53]. Following that rationale, the effectiveness of MST could, theoretically, be improved by increasing the stimulus' dose, which can be achieved by administering a larger number of pulses in the MST train. Early MST equipment employed pulse frequencies of 40 Hz to 50 Hz and train lengths of up to 8 seconds, for a maximum of 400 pulses. Specific equipment, capable of delivering stimulus at 100 Hz and for up to 10 seconds (1000 pulses), was developed in order to test such hypothesis [34]. Preclinical studies suggested that HD-MST delivered under such parameters could be safe from a cognitive point of view, with no histological damage, either compared to low-dose MST, Sham, or ECS [36, 37, 39].

MST shows a considerable advantage over ECT on its side effects profile and induced cognitive loss. Such difference was evident from the early preclinical studies comparing it to ECS, subjects showing significantly better cognitive scores after MST than ECS. On human subjects, reorientation time after MST ranges from 2 minutes to 8 minutes, while it takes from 18 minutes to 26 minutes after ECT, a considerable improvement. Other cognitive functions, such as retrograde and anterograde memory, language, and praxis, seem to be unaffected by MST. ECT, on the other hand, is notorious for inducing cognitive loss, which might be its most significant drawback.

Mood disorders are complex and varied in its symptoms and characteristics. All clinical studies revised here focused on depressive episodes and the majority of enlisted patients suffered from MDD, with very few cases of BD. Considering the challenges of treating depressive episodes in bipolar disorder and the low number of approved effective treatments, MST warrants further investigation as an alternative treatment for such cases. Furthermore, all studies which included BD patients included them on a sample majorly composed of MDD patients; no study so far has focused on bipolar depression specifically. Also, there are no reports of MST for manic episodes of BD. Considering ECT's record of effectiveness on manic states, the use of MST on such cases could be a focus for future research.

Being such a novel and still experimental technique, MST studies suffer from small samples and low statistical power. Also there is considerable variance in the MST technique employed by the researchers, which is unsurprising, considering that commercially available MST devices have only recently become available. However, there is little standardization of anesthetic methods, rating scales, cognitive assessments, and the protocol for ECT control. There is currently only one double-blind randomized clinical trial comparing the effectiveness and side effects of MST compared to ECT, and all the others are either case reports, open-label studies, or crossover studies. Future research, with larger samples, of double-blind design, and more consistent methods will allow for more statistic power and better understanding of the technique.

The findings reported on this review suggest that MST might be an effective and safe alternative for the treatment of mood disorders, specifically on treatment-resistant depression, with a safer and more tolerable side effects profile than the current first choice, ECT. However, further studies are necessary to improve the assessment of its potential effectiveness and expand current understanding of its mechanisms.

## Figures and Tables

**Table 1:** Clinical studies.

Author	Objective	Subjects	MST device and parameters	ECT device and parameters	Study design	Anesthetics	Cognitive and physiologic outcomes	Clinical outcomes
Lisanby et al., 2003 [22]	Assert the safety and feasibility of MST for MDD^1^	*N* = 10 (MDD)	50 Hz modified Magstim; first session titration for ST^2^, followed by sessions 0.5 ms PW^3^and 60 Hz at 100% output	Mecta 5000 Q; 0.5 ms pulse width; 9 patients RUL^4^-ECT x6 ST + 1 patient BL^5^-ECT 2.5x ST	Double-blind within subject crossover MST × ECT	Atropine 0.4 mg/Kg IV; methohexital 0.75 mg/Kg IV; succinylcholine 0.75 mg/Kg IV	MST superior to ECT on multiple cognitive domains; MST elicited shorter seizures	N/A
White et al., 2006 [43]	Evaluation of anesthetic aspects of MST	*N* = 20 (MDD)	50 Hz modified Magstim; first session titration for ST, followed by sessions at 1.3x ST	Mecta 5000 Q; BF^6^-ECT, 0.5 ms PW; 2.5x ST	Double-blind randomized trial MST × ECT	Etomidate 0.15–0.20 mg/Kg IV; succinylcholine 0.5–1.0 mg/Kg IV; glycopyrrolate 2.5 mcg/Kg IV; ketorolac 0.4 mg/Kg	MST resulted in lower variation on BIS^7^ and faster reorientation	ECT reduced HAM-D^8^ from 30 to 6; MST reduced HAM-D from 32 to 14 after 10–12 sessions
Kirov et al., 2008 [47]	Assessment of reorientation time after HD-MST^9^	*N* = 11	100 Hz modified Magstim; PW 0.34–0.4 ms, 10 s stimulus for all patients	Not specified	Double-blind crossover MST × ECT	Etomidate 0.15–0.3 mg/Kg IV; succinylcholine 0.5–1.0 mg/Kg IV	MST faster reorientation (7 : 12 min) compared to ECT (26 : 35 min)	N/A
Kayser et al., 2011 [44]	Effectiveness and safety of MST compared to ECT	*N* = 20 (16 MDD, 3 BP-II^10^ and 1 BP-I^11^)	100 Hz MagVenture MST MagPro; 0.37 ms PW;	Thymatron IV; 0.5 PW, RUL-ECT, 3x ST	Double-blind randomized trial MST × ECT	Propofol 1.5–2.5 mg/Kg IV; succinylcholine 1–1.5 mg/Kg IV	No cognitive loss on either group	MST: 60% response and 30% remission; ECT 40% response
Kayser et al., 2013 [48]	Assessment of cognitive and seizure characteristics of HD-MST and ECT	*N* = 7 (6 MDD, 1 BP-II)	100 Hz MagVenture MST MagPro;	Thymatron IV; 0.5 ms PW, 0.9 A, 30–120 Hz; 5 patients RUL-ECT 6x ST, 2 patients BL-ECTG 3x ST	Open-label, follow-up of MST after failure to respond to ECT	Propofol 1.0–1.5 mg/Kg IV; 1.0–1.5 mg/Kg succinylcholine IV	Shorter reorientation after MST; seizures similar, but shorter after MST	N/A
Hoy et al., 2013 [46]	Effects of MST on brain glucose metabolism	*N* = 10 (MDD)	100 Hz MagVenture MST; 400 pulses above ST	N/A	Open label	Propofol (mean dose 122.13 mg IV); succinylcholine (mean dose 53.61 mg IV)	Glucose metabolism increased in several areas	57% of response after treatment
Fitzgerald et al., 2013 [45]	Effectiveness and safety of MST	*N* = 13 (MDD)	100 Hz MagVenture MST MagPro; 10 s stimulus for first patient; 400 pulses above ST for all others	N/A	Open-label study	Propofol (mean dose 124.0 ± 24.1 mg IV); succinylcholine (mean dose 52.7 ± 12.2 mg IV)	Fast reorientation with patients reporting awakening under muscle relaxation	Five patients responded, two of which achieved remission
Polster et al., 2014 [49]	Compare acute memory retrieval of MST and ECT	*N* = 30 (20 MDD + 10 controls)	100 Hz MagVenture MST MagPro; suprathreshold stimulation; 2x week	Thymatron IV; 0.5 ms PW, RUL-ECT, 5x ST; 2x week	Open-label study	Propofol 1.5 mg/Kg IV; succinylcholine 1.0 mg/Kg V	Delayed recall disturbed after ECT but not after MST	N/A

*Notes.* ^1^ Major depressive disorder; ^2^seizure threshold; Hamilton Depression Rating Scale; ^3^pulse width; ^4^right unilateral electrodes; ^5^bitemporal electrodes; ^6^bifrontal electrodes; ^7^electroencephalographic bispectral index; ^8^Hamilton Depression Rating Scale; ^9^high-dose magnetic seizure therapy; ^10^bipolar disorder, Type II; ^11^bipolar disorder, Type I.
